# Genome-wide screening reveals opposing roles for central metabolism in planktonic and biofilm survival of *Salmonella* under combined limonene and heat treatment

**DOI:** 10.1016/j.bioflm.2026.100406

**Published:** 2026-10-29

**Authors:** Emma R. Holden, Silvia Guillén, Muhammad Yasir, Natalia Perez-Moral, Arjan Narbad, Alfredo Palop, Mark A. Webber, Enriqueta Garcia-Gutierrez

**Affiliations:** a Quadram Institute Bioscience, Norwich Research Park, Norwich, NR4 7UQ, UK; b Centre for Microbial Interactions, Norwich Research Park, Norwich, NR4 7UG, UK; c Departamento de Producción Animal y Ciencia de los Alimentos, Instituto Agroalimentario de Aragón - (IA2) - Universidad de Zaragoza-CITA, Zaragoza, Spain; d Department of Agronomic Engineering, Technical University of Cartagena, European University of Technology EUT+, Paseo Alfonso XIII, 48, 30203, Cartagena, Spain; e Instituto de Biotecnología Vegetal, Technical University of Cartagena, 30202, Cartagena, Spain; f University of East Anglia, Norwich Research Park, Norwich, Norfolk, NR4 7TJ, UK

**Keywords:** *Salmonella* Typhimurium, Biofilm, Limonene, Essential oil, Thermal inactivation, Nanoemulsion, TraDIS-Xpress, Food preservation, Food safety

## Abstract

*Salmonella enterica* serovar Typhimurium is a major cause of foodborne illness and remains a persistent challenge for food safety due to its ability to survive in both planktonic and biofilm-associated states along the farm-to-fork chain. While conventional control strategies rely on chemical preservatives and thermal treatments, increasing consumer demand for clean-label foods has driven interest in natural antimicrobials such as essential oils, of which limonene is a major citrus-derived component. However, the cellular mechanisms underlying *Salmonella*'s response to these strategies, and whether it differs between free and nanoemulsified formulations, or between planktonic and biofilm lifestyles, has not been defined. In this study, we applied an unbiased, transposon-directed insertion-site sequencing (TraDIS-*Xpress*) to systematically identify every gene and pathway contributing to the survival of *S.* Typhimurium when exposed to limonene in both free and nanoemulsion, across different inactivation temperatures and in both planktonic culture and established biofilms. We show that inactivation of genes involved in central metabolism and oxidative stress responses improves planktonic survival of heat treatment, but that this protective effect is lost under nanoemulsified limonene at 54 °C, a condition under which survival was not detected. Interestingly, these same genes and pathways were essential for the survival of established biofilms irrespective of heat or oil treatment, revealing that the genetic determinants of susceptibility invert between the two lifestyles. Together, these findings provide a genome-wide view of the genetic architecture underpinning *Salmonella* survival in response to limonene and heat and reveal that planktonic and biofilm cells have opposite vulnerabilities, meaning that effective decontamination strategies must be tailored to the physiological state in which *Salmonella* persists.

## 1 Introduction

*Salmonella enterica* serovar Typhimurium is a leading cause of foodborne infection worldwide and can persist throughout the farm-to-fork continuum by transitioning between planktonic and biofilm lifestyles on food surfaces, processing equipment, and host tissues. Salmonellosis is a substantial public health burden: non-typhoidal *Salmonella* infections are estimated to cause over 1.3 million illnesses and tens of thousands of hospitalisations annually in the United States alone, and globally *Salmonella* is responsible for 90 million cases of gastroenteritis and 155,000 deaths each year [1], [2]. These infections impose significant economic and healthcare costs, reflecting both direct treatment expenses and losses in food production, processing and distribution. One critical factor contributing to the persistence and widespread impact of *S. enterica* is its ability to form biofilms, structured microbial communities located in a self-produced extracellular matrix that enhances survival under environmental stress and resistance to disinfectants. Biofilm formation is a complex metabolic and regulatory process that involves an initial adhesion to surfaces followed by maturation with extracellular matrix production and eventual dispersal, and requires coordinated regulation of adhesion factors, motility, matrix biosynthesis, metabolism and stress responses. These attributes allow *Salmonella* to colonise food contact surfaces and processing equipment, where they become difficult to eliminate [3].

In industrial settings, controlling *Salmonella* contamination relies on a combination of good manufacturing practices and comprehensive sanitation strategies applied throughout the processing environment. Routine cleaning typically involves physical removal of residues, application of chemical sanitisers (quaternary ammonium compounds and chlorine-based agents) and equipment design that facilitates accessibility for thorough cleaning [4]. Thermal inactivation is also widely used to reduce microbial loads in foods, although biofilm-associated cells often display increased tolerance to heat compared with planktonic cells due to their matrix composition and different metabolic rates and stress responses [5].

In parallel to these conventional control measures, there is an increasing interest in replacing or complementing synthetic preservatives with natural antimicrobials, like essential oils, to meet consumers’ demand for clean label foods while maintaining microbiological safety [6]. Essential oils show broad-spectrum antimicrobial activity, but their high hydrophobicity, volatility, and variable stability have motivated the development of nanoemulsions to improve the bioavailability and interaction with bacteria and biofilm matrices [7], [8]. Much of this activity is due to individual components of these oils. Limonene, the principal monoterpene of citrus essential oils, is one such compound, generally recognised as safe and known to compromise bacterial membrane integrity [9].

Despite the increasing use of essential oils and thermal treatments in food preservation, the bacterial response they provoke remains poorly understood at the mechanistic level. The inactivation kinetics of limonene and heat against *Salmonella* have been characterised [7], [10], but the genetic determinants that allow cells to survive these combined chemical and thermal stresses have not been defined, nor it is known whether these determinants differ between free and nanoemulsions. This gap is particularly acute for biofilms, as matrix-embedded cells tolerate both antimicrobials and heat better than their planktonic counterparts [11]. Whether this reflects a distinct set of survival genes, therefore, a distinct set of targets, is unknown. Transcriptomic studies can reveal gene expression changes but cannot distinguish genes that are simply responsive from those contributing to survival. A genome-wide loss-of-function approach is needed to make this distinction. Resolving this would allow *Salmonella* to be targeted more effectively according to its lifestyle rather than treated as a single uniform population.

Transposon-directed insertion-site sequencing (TraDIS) addresses this by assaying the contribution of every gene to survival simultaneously: a dense library of transposon mutants is exposed to a condition of interest, and the relative depletion or enrichment of insertions in each gene reports its effect on fitness [12]. The TraDIS-*Xpress* adaptation additionally incorporates an outward-facing promoter within the transposon, allowing changes in gene expression, not only gene inactivation, to be captured, and thereby resolving genes whose altered dosage affects survival [13]. The approach has previously been applied to *Salmonella* to identify determinants of preservative and antibiotic susceptibility [14], [15], making it well suited to identifying the genes underpinning survival under combined chemical and thermal stress across different bacterial lifestyles.

Here, we map the genetic basis of *Salmonella* survival under combined limonene and heat treatment across both lifestyles. Using the transposon-directed insertion-site sequencing screen TraDIS-*Xpress*, we identified conditionally essential genes underpinning survival of free and nanoemulsified limonene, applied at inactivation temperatures of 54 °C or 56 °C against a 37 °C control to mimic industrial cleaning protocols, in planktonic culture and established biofilms in parallel. To our knowledge, this is the first study to define the genome-wide determinants of essential-oil and heat survival in *Salmonella*, and the first to compare them between formulations and between planktonic and biofilm states. This parallel design reveals lifestyle-specific vulnerabilities that could inform the design of preservation schemes combining essential oils, nanoemulsions and thermal processing.

## 2 Methods

### 2.1 Preparation of nanoemulsion

An oil-in-water nanoemulsion of limonene was prepared following a previous protocol with modifications, using a Branson Digital Sonifier (UK) [10]. Briefly, the continuous aqueous phase was prepared by mixing 5.5 mL of polyethylene glycol 400 (Sigma-Aldrich, UK) with 9.9 mL of distilled water. Then, 3.2 mL of R-(+)-limonene (97%, Sigma-Aldrich, Spain) and 2.4 mL of Tween 80 (Sigma-Aldrich, UK) were added. The final emulsion was obtained when the combined aqueous and oil phases were sonicated using a probe sonicator at 400W, 100% amplitude, in 3 cycles of 1 min followed by 1 min to cool.

The particle size distribution of the nanoemulsions was measured by laser diffraction using a Beckman-Coulter LS 13,320 XR laser diffraction particle sizer analyser, equipped with a Universal Liquid Module (ULM). The emulsions were diluted with distilled water prior to analysis to prevent back-scattering. The particle and water refractive indices employed were 1.473 and 1.333 respectively. Each measurement was performed in triplicate.

The mean particle size of the nanoemulsion preparation used in this study was 0.769 μm calculated as the arithmetic mean of three consecutive runs, and good repeatability of the measurements (coefficient of variation CV = 0.49%) (Supplementary Fig. 1). The particle diameters spanned from 0.06 to 4 μm and the standard deviation of the particle size distribution was 0.611 μm, suggesting a moderately broad size distribution. The particle size distribution curve showed a dominant population within the submicron range, confirming the formation of a fine emulsion system. No secondary peaks corresponding to large droplets or aggregates were observed within the measured size range indicating the absence of significant droplet coalescence. Overall, the results confirm that the prepared formulation exhibits a submicron particle size with consistent measurements across repeated runs.

### 2.2 Planktonic set up and essential oil and temperature treatments

*S*. *enterica* serovar Typhimurium 14028S was used throughout this study. The transposon mutant library used in this work was previously described by Ref. [16], [16]). The transposon used in this library contains a *tac* promoter that when induced allows simultaneous assessment of loss- and gain-of-function effects and extending analysis to essential genes that cannot be inactivated in conventional mutagenesis screens [13]. The library comprises approximately 500,000 unique transposon mutants, providing near-saturating genome coverage.

Approximately 10^7^ CFU/mL of library was added to 5 mL LB broth without salt supplemented with 1 mM Isopropyl β-d-1-thiogalactopyranoside (IPTG, Formedium, UK) in sterile 5 mL eppendorfs. A sub-lethal concentration for limonene against *Salmonella* (0.2 μL/mL) was added to each tube [17], required to retain a viable, diverse mutant population for TraDIS-*Xpress* analysis while imposing selective pressure. This concentration (≈0.17 mg/mL) is below reported MIC values for limonene against Salmonella (0.42-1.6 mg/mL), ensuring a sub-inhibitory exposure. The volume of nanoemulsion was adjusted to deliver the same final limonene concentration as the free oil treatment. Tubes with limonene, nanoemulsion and untreated controls were placed in a water bath at 54 °C for 26 min, 56 °C for 9 min or 37 °C for 1 h. These thermal conditions were selected as sub-lethal exposures based on preliminary experiments, designed to cause measurable inactivation while retaining sufficient viable cells for TraDIS-Xpress analysis. After the treatments, media was removed to eliminate the presence of the limonene, replaced with 1 mL of fresh LB, and incubated at 37 °C for 4 h to allow cells to recover and repopulate. Cells were then pelleted and stored at −20 °C until DNA extraction.

### 2.3 Biofilm set up and essential oil and temperature treatments

A total of 30 sterile glass beads (5 mm, Merck) were placed in each well of a 6-well plate (Corning, UK) containing 5 mL of LB broth without salt, 1 mM IPTG and approximately 10^7^ CFU/mL of transposon mutant library. Plates were sealed with gas-permeable Breathe-Easy® membranes (Sigma-Aldrich, UK) to prevent evaporation while maintaining aerobic conditions, and incubated at 30 °C for 48 h to form established biofilms on the beads. Treatments were applied similarly to the planktonic conditions: a total of 1 μL (0.2 μL/mL) of limonene or nanoemulsion was added to each well and the plates were placed in a water bath at 54 °C for 26 min, at 56 °C for 9 min and at 37 °C for 1 h. After the treatments, media was removed to eliminate the presence of the limonene, and was replaced with 1 mL of fresh LB, incubated at 37 °C for 4 h to allow time for mutant replication. Cells were removed from the glass beads by vortexing in PBS and were then pelleted and stored at −20 °C until DNA extraction.

### 2.4 DNA extraction and TraDIS-*Xpress* sequencing library preparation

DNA was extracted using a Maxwell® Robot (Promega, UK) with Maxwell® RSC PureFood GMO and Authentication Kit AS1600 (Promega, UK), following manufacturer's protocols. DNA concentration was measured using Qubit 2.0 Fluorometer (Thermo Fisher Scientific, UK) and normalised for library preparation. Libraries were prepared following the protocol outlined by Holden et al. [14] to amplify and isolate DNA fragments containing the transposon. DNA fragment length was quantified using a Tapestation (Agilent) and sequenced on a NextSeq500 using the NextSeq 500/550 High Output Kit v2.5 with 75 cycles. QuaTraDIS (version 1.4) (https://github.com/quadram-institute-bioscience/QuaTradis) was used to map fastq files to a *S.* Typhimurium reference genome (CP001363, modified to include the chromosomally-integrated *tac* promoter) and determine statistically significant differences in insertion frequencies between conditions. A set of default cut-offs for significance and number of reads were applied (q-value < 0.05, logFC ≥2, logCPM ≥4) to create a list of candidate genes.

### 2.5 Validation of TraDIS-*Xpress* results with single gene deletion mutants

Single gene deletion mutants of *crp* and *cyaA* (made for previously published work [16], [18] were treated to the same exposure conditions in planktonic culture and established biofilms as previously described for the TraDIS-*Xpress* experiments. Following the exposure, cells were removed from the glass beads by vortexing in PBS. To calculate CFU/mL recovered following each exposure, recovered cells from the planktonic and biofilm exposure conditions were serial diluted and 10 μL of each dilution was spotted onto LB agar, incubated overnight at 37 °C and counted the following day. This was repeated for two biological and three technical replicates.

### 2.6 Intracellular ROS quantification by DCFH-DA fluorescence

Intracellular reactive oxygen species were quantified using the cell-permeable probe 2′,7′-dichlorodihydrofluorescein diacetate (Sigma-Aldrich, Spain). Overnight cultures of *S*. Typhimurium 14028S were diluted 1:100 in LB broth without NaCl and grown to mid-exponential phase (OD_600_ ∼0.5). Cells were harvested by centrifugation (5000 × g, 5 min), washed once with PBS, and resuspended in PBS containing 20 μM DCFH-DA. Suspensions were incubated at 37 °C for 30 min in the dark to allow probe uptake and intracellular de-esterification. After loading, cells were washed twice with PBS to remove extracellular probe and resuspended in LB broth.

Aliquots (200 μL) were transferred to a black 96-well flat-bottom microplate (Sigma-Aldrich, Spain) and exposed to: (i) 37 °C for 26 min (untreated control); (ii) 37 °C with 0.2 μL/mL free (unformulated) limonene for 26 min; (iii) 54 °C for 26 min; and (iv) 54 °C with 0.2 μL/mL free limonene for 26 min; (v) 56 °C for 9 min; and (vi) 56 °C with 0.2 μL/mL free limonene for 9 min. Free limonene was used to avoid confounding by nanoemulsion excipients. Positive controls of 0.1 mM H_2_O_2_ at 37 °C and LB + 20 μM DCFH-DA without cells were included to validate probe responsiveness and detection. After treatment, cells were pelleted by centrifugation (5000 × g, 5 min), resuspended in 200 μL PBS, and fluorescence was measured using a Tecan Infinite 200 PRO microplate reader (excitation 485 nm, emission 530 nm, bandwidth 9/20 nm, gain 100 manual, 25 flashes). Two independent biological replicates were performed, each with three technical replicates. Data were analysed by one-way ANOVA followed by Tukey's HSD post-hoc test using GraphPad Prism 8 (GraphPad Software, San Diego, CA, USA). Differences were considered significant at p < 0.05.

## 3 Results

### 3.1 Central metabolism and oxidative stress genes are principal determinants of planktonic survival under limonene and heat treatment

We used TraDIS-*Xpress*, a large-scale transposon mutagenesis approach, to investigate the effect of essential oil and heat treatments on *Salmonella* survival. A library of approximately 500,000 unique mutants was exposed to limonene in two preparations (oil and nanoemulsion) in planktonic culture and in established biofilms at three temperatures: 54 °C for 26 min, 56 °C for 9 min or 37 °C for 1 h. After the treatments, media was removed to eliminate the presence of the limonene and replaced with sterile LB broth. Mutant abundance following these exposures was quantified via sequencing and compared to untreated controls to investigate the contribution of each gene to *Salmonella* survival in each condition. Across all conditions, there were 196 unique genes identified to affect survival (Supplementary Table 1, Fig. 1).

**Fig. 1:**
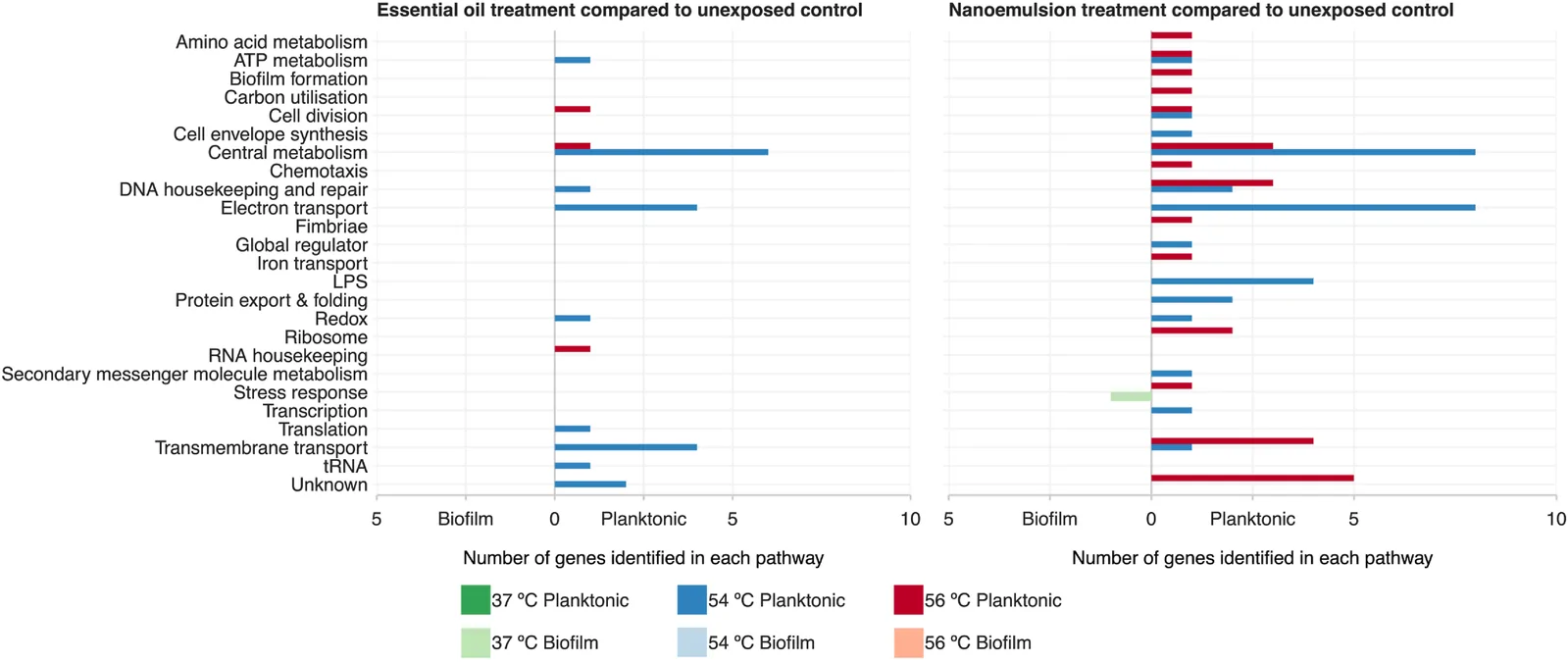
Pathways involved in survival of *S*. Typhimurium following exposure to essential oil or nanoemulsions at 37, 54 or 56 °C in planktonic cultures or established biofilms.

Over a quarter (29%) of genes identified by TraDIS-*Xpress* across all exposure conditions had direct roles in central metabolism, energy generation and oxidative stress responses (Fig. 1), suggesting a role for reactive oxygen species in cell inactivation. We found increasing temperatures alone reduced the essentiality of genes involved in central metabolism in planktonic cultures, consistent with heat-induced oxidative stress. Inactivation of regulators of central metabolism *cra*, *crp* and *cyaA* improved survival at 54 °C, with a 10.0, 6.2 and 5.3 log-fold increase in mutant abundance seen relative to 37 °C, respectively (Fig. 2a and b,c).

**Fig. 2:**
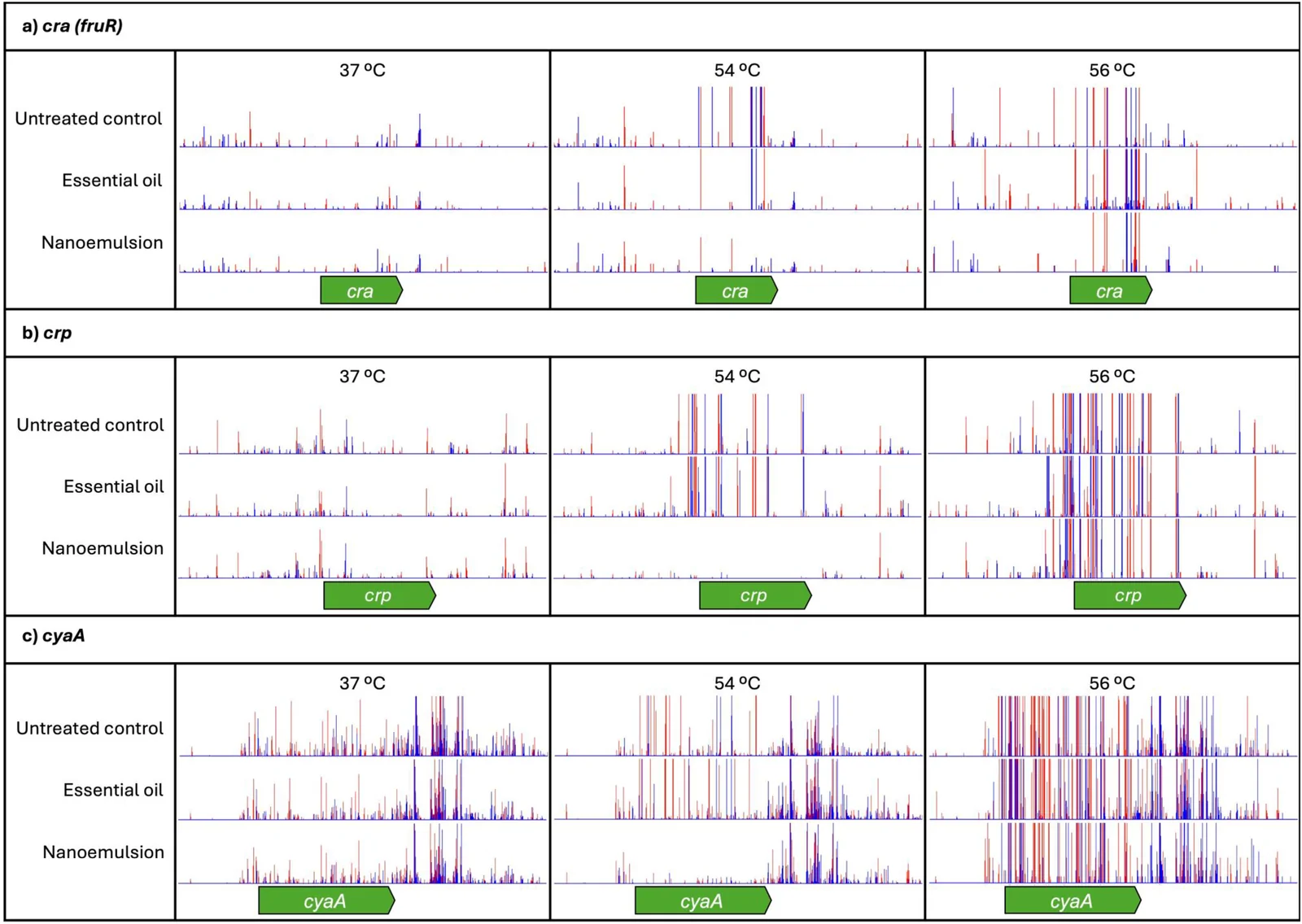
Transposon insertion loci and frequency around a) *cra* (fruR), b) *crp* and c) *cyaA* in planktonic cultures of *S*. Typhimurium treated with two different essential oil preparations at three temperatures. Red lines indicate insertions in a 5′ to 3′ orientation and blue lines a 3′ to 5′ orientation. One of two independent replicates is shown. (For interpretation of the references to colour in this figure legend, the reader is referred to the Web version of this article.)

When treated with limonene at 54 °C, genes involved in central metabolism improved survival relative to untreated culture. There were 3.2 log-fold fewer mutants in *cra* in planktonic cultures treated with oil and 9.4 log-fold fewer when treated with nanoemulsions compared to untreated controls at 54 °C (Fig. 2a). This was also true for *sdhC* encoding a major subunit of succiante::quinone oxidoreductase involved in energy generation in the TCA cycle (6.1 log-fold reduction in oil-exposed and 10.3 log-fold reduction in nanoemulsion-exposed) and *nuoG* encoding a subunit of NADH dehydrogenase I involved in energy generation via the electron transport chain (4.2 log-fold reduction in limonene-exposed and 4.6 log-fold reduction in nanoemulsion-exposed) [19]. This suggests genes involved in central metabolism and the management of oxidative stress are important for survival following exposure to combined limonene and heat treatments.

At 56 °C, only two genes crucial for survival were shared between both limonene preparation exposures; *mioC* involved in regulating cell division (10.7 and 10.2 log-fold fewer insertions with exposed to oil or nanoemulsion, respectively) and *sucB* involved in the TCA cycle (10.0 and 9.6 log-fold fewer insertions with exposed to oil or nanoemulsion, respectively). This highlights the importance of DNA housekeeping and central metabolism in survival under combined nanoemulsion and heat exposure.

To validate the importance of central metabolism to survival in the exposure conditions, we constructed gene deletion mutants in *crp* and *cyaA* and quantified viable CFU/mL of planktonic culture after exposure to limonene and nanoemulsions at 37 °C, 54 °C and 56 °C (Fig. 3). Relative to the wild type, deletion of *crp* or *cyaA* did not significantly affect survival when exposed to limonene or nanoemulsion at 37 °C (Two-way ANOVA, *p* = 0.9). This supports our findings from the TraDIS-*Xpress* screen indicating these genes do not affect survival at 37 °C.

**Fig. 3:**
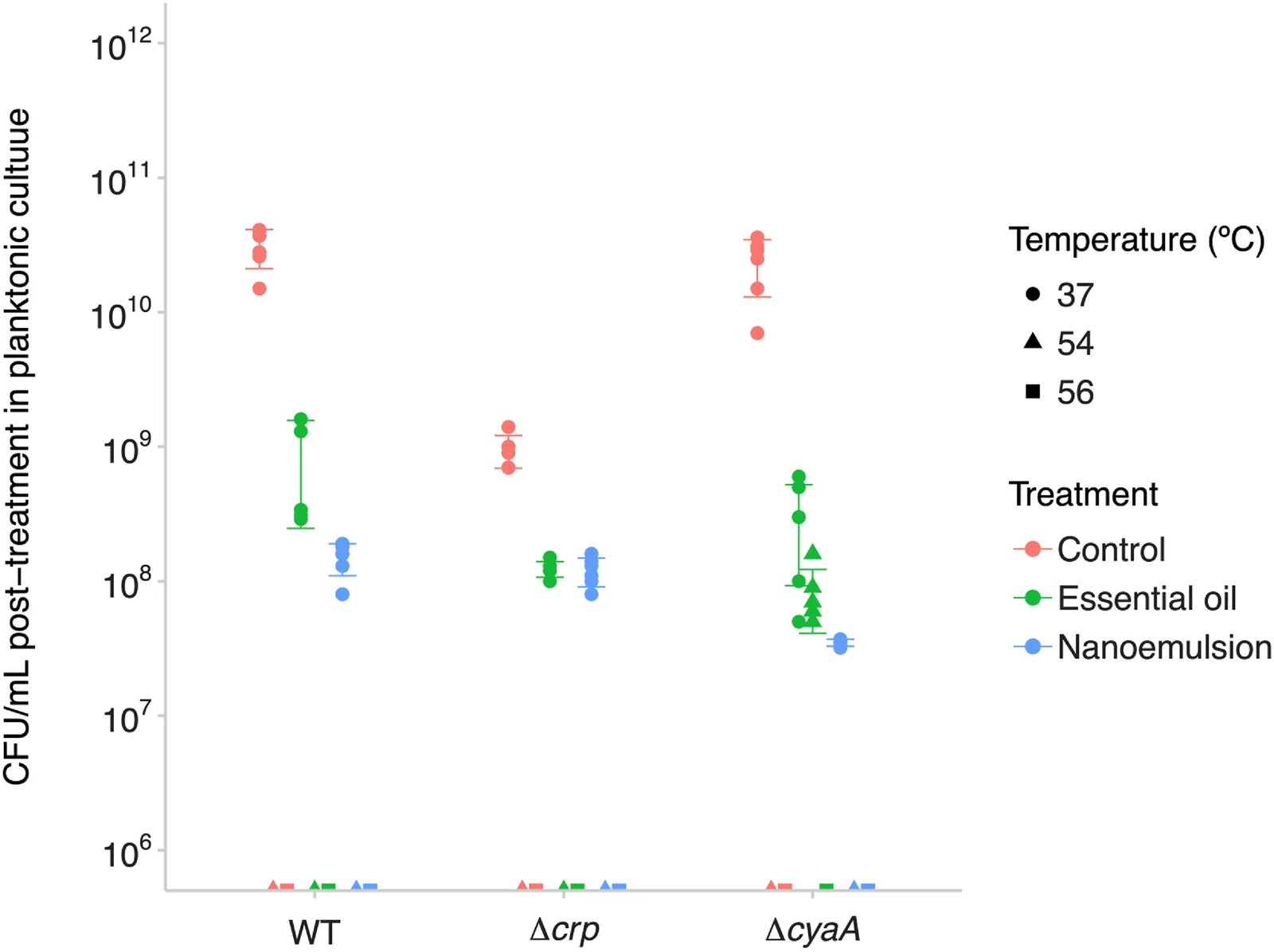
CFU/mL in planktonic cultures of wild type *S.* Typhimurium and single gene deletion mutants following exposure to limonene and nanoemulsion (and an untreated control) at 37, 54 and 56 °C. Points show two biological and three technical replicates and error bars show 1 standard deviation. Values of zero are displayed at the base of the y-axis as they cannot be represented on a log scale.

Based on the findings from the TraDIS-*Xpress* screen, we predicted these mutants would have improved survival at 54 and 56 °C relative to the wild type. At 54 °C, surviving cells could only be recovered from a *cyaA* deletion mutant treated with limonene (Fig. 3). This validates our findings from the TraDIS-*Xpress* screen that inactivation of *cyaA* improves survival in these conditions. No cells could be recovered from any cultures treated at 56 °C.

### 3.2 Limonene preparations affect *Salmonella* survival differently in planktonic culture

There were differences in gene essentiality between each limonene preparation at 54 °C, where inactivation of genes involved in central metabolism improved survival in untreated culture and limonene-treated cultures relative to at 37 °C, but had no significant effect on the survival of nanoemulsion-treated cultures. Following inactivation of *crp* and *cyaA* (involved in global regulation of central metabolism, Fig. 2b and c), there were 3.9 and 7.1 log-fold fewer mutants in these genes respectively following exposure to nanoemulsions relative to untreated controls at 54 °C. At 56 °C there were no differences in gene essentiality seen between the two oil preparation treatments.

The difference in conditional essentiality of metabolic genes between the oil preparations at 54 °C suggests inactivation of these genes does not confer a fitness benefit in these conditions. This may indicate the cell is not under the same degree of oxidative stress and that other mechanisms are responsible for cell inactivation. We identified genes involved in nitrite and nitrate reductase systems were important for *Salmonella* survival when treated with nanoemulsions at 54 °C: there was a reduction in mutant abundance in *narH* (2.0 log-fold) and *napC* (2.3 log-fold), relative to an untreated control. This suggests the nanoemulsion treatment might impose additional stress responses beyond those seen with free limonene alone in *Salmonella*.

Further differences between oil and nanoemulsion preparations were seen in the essentiality of genes involved in outer membrane composition. Inactivation of 22 genes involved in LPS biosynthesis and 5 genes involved in synthesis of enterobacterial common antigen (ECA) improved survival when treated with nanoemulsion at 54 °C relative to 37 °C. Genes encoding outer membrane chaperone SurA and thiol:disulfide oxidoreductase DsbA, both with established roles in outer membrane protein folding, also improved fitness when inactivated (2.7 and 2.3 log-fold more insertions, respectively) in nanoemulsion-treated conditions relative to an untreated control at 54 °C. This suggests nanoemulsions have a larger effect on membrane composition relative to oil.

Oxidative stress and nitrosative stress are known to induce DNA damage and our data shows a crucial role for *alkB* (catalyses non-mutagenic DNA repair, 10.9 log-fold fewer mutants) and *holD* (DNA polymerase III subunit, 9.8 log-fold fewer mutants) following exposure to nanoemulsions at 56 °C, relative to an untreated control. Overall, these differences in gene essentiality between oil-treated and nanoemulsion-treated cultures suggest the two preparations affect survival of *Salmonella* in distinct fashions.

### 3.3 Regulation of central metabolism is essential for biofilm formation in *Salmonella*

We saw differences in mutant survival following heat and oil exposure between established biofilms and planktonic culture. The most striking finding was that the same genes involved in central metabolism that affected mutant survival in planktonic culture were essential for biofilm survival across all conditions (Fig. 4). Across all conditions, there were between 3.8 and 12.3 log-fold fewer insertions in *crp*, between 2.6 and 9.5 log-fold fewer insertions in *cyaA* and between 3.1 and 15.1 log-fold fewer insertions in *cra* in biofilm conditions relative to planktonic conditions (Fig. 4).

**Fig. 4:**
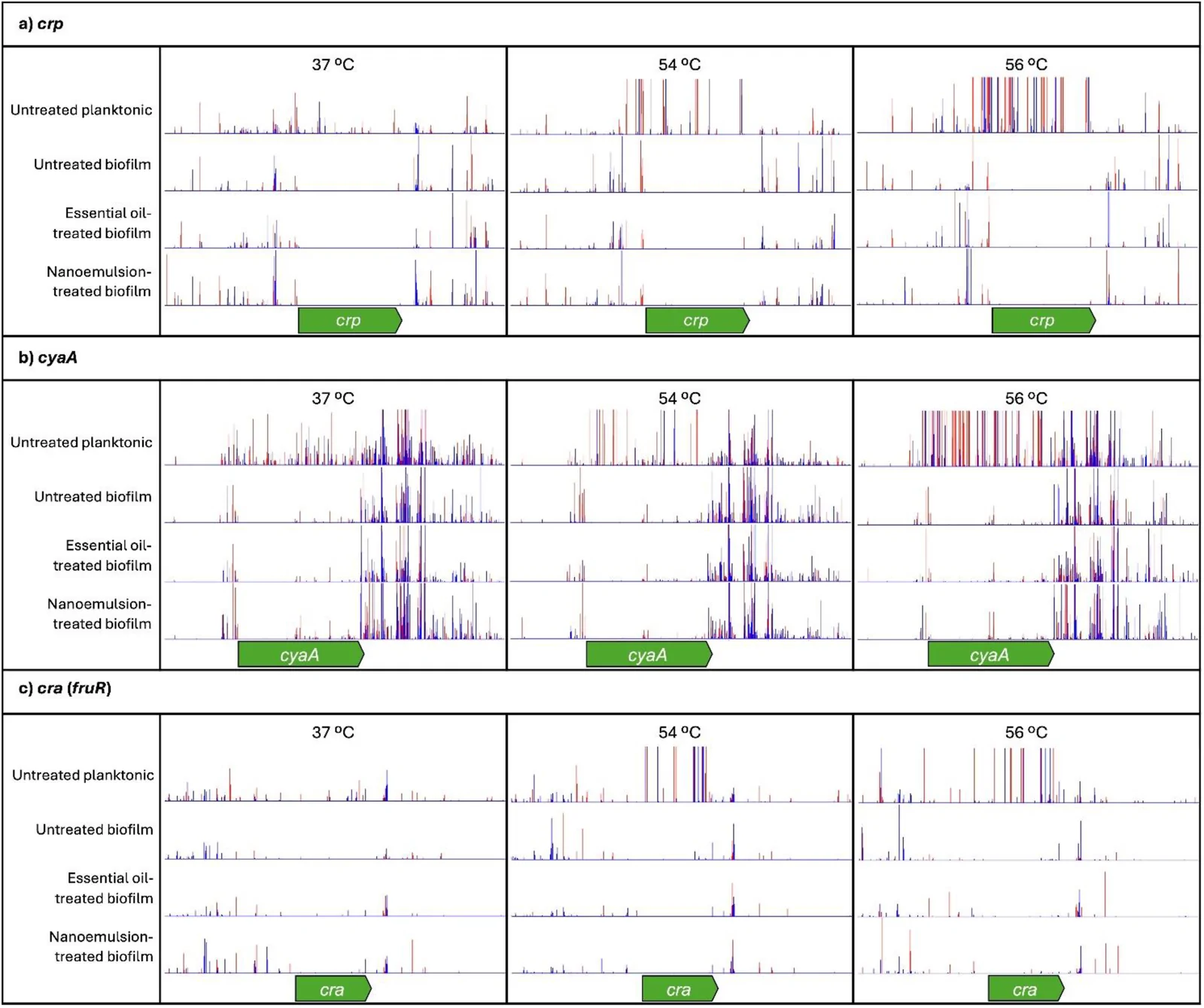
Transposon insertion loci and frequency around a) *crp*, b) *cyaA* and c) *cra* (*fruR*) in biofilm cultures of *S*. Typhimurium treated with two different essential oil preparations at three temperatures. Red lines indicate insertions in a 5′ to 3′ orientation and blue lines a 3′ to 5′ orientation. One of two independent replicates is shown. (For interpretation of the references to colour in this figure legend, the reader is referred to the Web version of this article.)

Genes where expression is essential for survival in one condition and harmful in another are potential targets for combination treatments that work in synergy to reduce bacterial load. We aimed to investigate this by growing biofilms in gene deletion mutants of *crp* or *cyaA* treated with oil or nanoemulsion at different temperatures and quantifying mutant viability after treatment. We believed the deletion mutants would be more able to form a biofilm in isogenic cultures relative to in the transposon mutant pooled library, however as predicted by the TraDIS-*Xpress* screen these genes are essential for biofilm formation and deletion mutants could not form a biofilm.

We found only one gene where its essentiality differed between treated and untreated biofilms. We found 4.3 log-fold more insertions upstream of *hyaD* (STM14_2164) in biofilms treated with nanoemulsions at 37 °C relative to untreated controls. This encodes a protease involved in the maturation of hydrogenase 1 and our data suggests increased expression improves survival in biofilms treated with nanoemulsions. Hydrogenase 1 catalyses the reduction of oxygen to water thereby preventing the formation of free oxygen radicals [20]. This supports our hypothesis that oil treatments inactivate *Salmonella* through generation of free oxygen radials. We found no differences in gene essentiality between untreated biofilms and those treated with oils in either preparation at 54 °C or 56 °C.

## 4 Discussion

This study defines the genetic basis of *Salmonella* survival under limonene and heat treatment in planktonic cultures and established biofilms, revealing that central metabolism acts in opposite directions depending on bacterial lifestyle, with genes whose inactivation improved planktonic survival of heat were, in biofilms, essential for survival irrespective of treatment. Across both lifestyles, genes involved in central metabolism, energy generation and oxidative stress responses dominated the survival response, consistent with oxidative damage as a route of cell inactivation. This interpretation is strongly supported by recent work in *Salmonella* itself: central carbon metabolism and the respiratory chain drive endogenous production of reactive oxygen species, and disabling ATP synthase raises metabolic flux, NAD(P)H and ROS to increase killing by fluoroquinolones [21]. The same effect has been reported for heat and essential-oil exposure in other bacteria [22], [23].

The data generated in this study points towards oxidative damage as a route of killing. Under heat alone, loss of the central-metabolic regulators *cra*, *crp* and *cyaA* improved survival, consistent with reduced metabolic flux lowering endogenous reactive oxygen species. Under combined limonene and heat, mutants in *nuoG* (Complex I) and *sdhC* (Complex II) were strongly depleted, implicating the respiratory electron transport chain, for which ubiquinone is the shared electron acceptor. Both complexes have been directly implicated in ROS generation in *Salmonella*, and inactivation of the *nuo* operon and *sdhCDAB* reduces oxidative killing [21], consistent with their depletion in our screen marking them as determinants of survival under oxidative attack. A comparable inversion in the value of respiratory metabolism has been reported for intracellular *Salmonella*: deletion of ubiquinone biosynthesis increases recovery from macrophages, where the respiratory burst has already inactivated the chain, yet attenuates replication in epithelial cells [24]. That a functional chain is protective under heat alone but required once limonene is present is consistent with it acting as the dominant endogenous source of reactive oxygen species during thermal stress, while becoming a determinant of survival when membrane integrity is compromised. These convergent genetic signatures, together with the direct demonstration that these same genes govern ROS production in *Salmonella*, support oxidative damage as a route of inactivation. To validate this interpretation, we measured intracellular ROS using a DCFH-DA fluorescence assay in *S*. Typhimurium 14028S under planktonic conditions matching those of the TraDIS-Xpress screen (Supplementary Fig. S2). Cells exposed to 54 °C showed higher intracellular ROS compared to the 37 °C untreated control, while free limonene alone did not increase ROS levels and the combination of 54 °C and limonene did not produce more ROS than temperature alone. At 56 °C, fluorescence dropped below the untreated control. Given that no cells were recovered at this temperature in the CFU-based validation experiments, this could be consistent with extensive cell lysis causing membrane disruption and loss of the intracellular probe.

Cyclic AMP (synthesised by adenylate cyclase encoded by *cyaA*) and the cyclic AMP receptor protein encoded by *crp* were found in our screen to negatively affect survival of untreated or oil-treated planktonic cultures of *Salmonella* at 54 °C, but had no effect on the survival on nanoemulsion-treated cultures at 54 °C. Both these genes have previously been implicated in negatively regulating persister cell formation [25]. Persistence is a highly regulated reversible physiological state whereby bacterial metabolism is slowed and cells become more tolerant to high concentrations of antimicrobial compounds [26]. Inactivation mutants of *cyaA* and *crp* may therefore have improved survival in our screen due to uninhibited persister cell formation. In our follow-up experiments with isogenic cultures of gene deletion mutants, we could not recover wild type or mutant *Salmonella* treated with nanoemulsions at 54 °C, but we could recover a *cyaA* deletion mutant following treatment with oil at 54 °C, consistent with combined heat and essential oil treatment inducing persister cell formation. That this pattern was not fully reproduced across all isogenic mutants is expected, as pooled TraDIS-*Xpress* and isogenic deletion assays interrogate fitness at different scales, the pooled library detecting fitness differences below the resolution of competition between isogenic strains at a defined density.

Previous work in *E. coli* found cultures that had undergone a 45 °C pre-treatment had improved survival to meropenem exposure relative to those that did not undergo a heat treatment, suggesting heat may pre-condition the cell to have increased tolerance to antimicrobials [27]. This has concerning implications for the use of combined heat and essential oil treatments in the food chain. That *cyaA* and *crp* inactivation improved survival under oil but not nanoemulsion at 54 °C is consistent with the nanoemulsion imposing a lethality that exceeds the protection persistence can confer, as no wild-type or mutant cells were recovered from nanoemulsion treatment at 54 °C, leaving no window in which a persistence-promoting mutation could register a benefit. Persister cell formation is common in biofilms [28], however we found both *cyaA* and *crp* are essential genes in treated and untreated biofilms and their inactivation is only tolerated in planktonic cultures. Further work is needed to understand whether combined heat and essential oil treatments induce persister formation to a degree that could affect food safety. It should be noted that the sub-inhibitory concentration used (≈0.17 mg/mL) was confirmed sub-lethal at 37 °C by our recovery data and became lethal only in combination with heat, consistent with the intended selective window across all three temperatures tested. As well as physiological changes, future work should determine whether genetic changes in regulators of central metabolism naturally arise in food production environments in response to decontamination.

As persistence, heat and essential oil treatments are reported as inducers of the viable but non-culturable (VBNC) state in *Salmonella* defined as a survival strategy in which bacteria remain metabolically active and alive yet fail to grow on routine culture media [29]. The CFU-based validation experiments used in this study cannot distinguish between killing and VBNC, and the observation that no cells were recovered at 56 °C could reflect VBNC induction rather than killing. This does not affect the TraDIS-Xpress findings, as cells that get into the VBNC state do not replicate during the recovery period and are functionally equivalent to dead cells in the screening. However, if treatments induce VBNC entry rather than killing, this has implications for food safety, as VBNC cells could reactivate under favourable conditions. Future work should incorporate viability staining or molecular methods to distinguish VBNC cells from dead cells following combined limonene and heat treatment.

Both LPS and ECA are glycolipids expressed on the outer membrane and have been previously suggested to be a potential drug target for citrus-derived essential oils, such as limonene [30]. Our data shows inactivation of LPS and ECA biosynthesis genes improves mutant survival in conditions exposed to a combination of nanoemulsions and heat, supporting the hypothesis that disruption of this potential drug target makes *Salmonella* less susceptible to the treatment. We cannot exclude that this fitness gain reflects general envelope remodelling rather than a specific interaction between nanoemulsion components and LPS. However, the restriction of this effect to nanoemulsion treatment at 54 °C, and its absence under free oil or other temperatures, argues against a purely nonspecific mechanism. The importance of LPS to *Salmonella* survival under combined antimicrobial and thermal stress has precedent: a genome-wide screen for survival in egg white at 42 °C found that the majority of required genes were in LPS biosynthesis [31], and Tn-seq of *Salmonella* survival on low-moisture foods likewise recovered LPS biosynthesis, DNA repair and stringent-response genes [32]. That LPS biosynthesis is required for survival in those settings, but its inactivation improves survival under nanoemulsion and heat here indicates that the direction of the LPS contribution is stress-specific rather than fixed. We only see the effect of LPS on survival when *Salmonella* is treated with nanoemulsions at 54 °C and in no other treatment conditions, suggesting that this contributes to increased susceptibility but is not the primary mechanism of killing. LPS has a well-established role in membrane permeability [33] and further research should focus on characterising if susceptibility is mediated through this route. The enhanced lethality of the nanoemulsified formulation might reflect improved bioavailability of limonene by increasing surface contact with bacterial cells, sustained release from emulsion droplets, and surfactant-mediated membrane perturbation, all of which could sensitise cells to concurrent thermal stress. A limitation of this study is the absence of a vehicle-only control (surfactant and polymer without limonene), which would distinguish effects of the formulation matrix from limonene itself. However, the inclusion of a free limonene treatment at the same concentration provides a partial control for this confounding, as gene fitness effects shared between both formulations can be attributed to limonene, while effects unique to the nanoemulsion treatment may reflect contributions from the vehicle matrix. The broad particle-size distribution of the nanoemulsion (0.06-4 μm) also means that bacterial cells were exposed to a heterogeneous droplet population. We cannot exclude that variable droplet size contributes to the phenotypic differences observed between free and emulsified limonene. Future work should include vehicle controls and dynamic light scattering characterisation to resolve these contributions.

The depletion of *narH* and *napC* under nanoemulsion treatment at 56 °C is consistent with nitrosative stress, although we did not measure nitrogen-radical species directly and this remains a hypothesis for future work. Oxidative and nitrosative stresses may co-occur during antimicrobial challenge, and their interaction can intensify Salmonella killing, in part through formation of the bactericidal oxidant peroxynitrite. This mechanism may therefore contribute to the enhanced lethality of the nanoemulsified formulation observed at higher temperatures [34]. Central metabolism was crucial to the survival of *Salmonella* biofilms, irrespective of treatment. We previously used the same TraDIS-*Xpress* whole genome screen to identify genes involved in *Salmonella* biofilm formation over time. This also identified genes involved in central metabolism as essential for biofilm formation and development over time, such as *cra*, *cyaA* and the *nuo* operon. Our previous work was cultured continuously at 30 °C, where *crp* was found to be essential for both planktonic and biofilm survival and was therefore not identified as essential for biofilm formation specifically. This suggests *crp* essentiality is temperature-dependent, *crp* was essential at 30 °C in our prior biofilm screen, dispensable at 37 °C in planktonic culture, and beneficial when inactivated at 54 °C, consistent with temperature-dependent essentiality across three independent conditions. Temperature-dependent essentiality of *crp* has previously been described in *Yersinia pseudotuberculosis* [35], but further work is needed in *Salmonella* and other Enterobacteriaceae to determine the extent to which this is conserved. Understanding the essentiality of genes involved in central metabolism could be key for the development of antimicrobial and anti-biofilm agents for controlling contamination in the food chain.

We identified increased expression of *hyaD* improved survival in *Salmonella* biofilms treated with nanoemulsions at 37 °C relative to untreated controls. This was the only gene found to affect survival in treated biofilms relative to untreated biofilms. Hydrogenase 1, encoded in the *hya* operon, reduces oxygen and can limit the formation of reactive oxygen species, so increased *hyaD* expression improving survival is consistent with the oxidative mechanism proposed above. This highlights the power of TraDIS-*Xpress* and the use of outwards facing promoters to investigate changes in gene expression alongside gene presence/absence, as this would not have been identified using conventional transposon sequencing approaches. TraDIS-*Xpress* has been used previously to investigate the effects of common food preservatives against *S*. Typhimurium [14] and biofilm formation on abiotic surfaces [16] and fresh produce [36], as well as the differences in antibiotic activity in planktonic and biofilm cultures [15]. All experiments were conducted in laboratory media on glass beads, which do not capture the organic load, surface heterogeneity or mixed-species communities of food-processing environments. Biofilm architecture and matrix composition can vary with the substrate material, which ultimately can influence the penetration of antimicrobial compounds and therefore the relative importance of individual genes identified here. Nevertheless, the role of genes involved in central metabolism in biofilm survival is consistent with fundamental biofilm physiology rather than a surface-specific effect. Future work should extend essential oil and heat treatments to *Salmonella* biofilms on a range of biotic and abiotic surfaces, as well as investigating activity in soiled environments, which could reduce the potency of these chemicals [37].

An important consideration is whether pH-mediated tolerance contributes to survival under the conditions tested. *Salmonella* possesses multiple acid tolerance response (ATR) systems, and pH is known to modulate both thermal resistance and susceptibility to essential oils. However, none of the canonical ATR genes were identified among the 196 genes affecting survival in our screen: the glutamate decarboxylase system (*gadABCE*), the arginine decarboxylase system (*adiAC*), acid stress chaperones (*hdeAB*), and key ATR regulators (*phoP/Q*, *evgA/S*) were all absent. As TraDIS-Xpress is an unbiased, genome-wide approach, any gene contributing to survival would be detected regardless of prior hypothesis. The absence of ATR genes indicates that pH-mediated acid tolerance was not a dominant survival mechanism under these conditions. While *rpoS* was identified, its inactivation improved planktonic survival at 54 °C (log_2_FC = +3.7), consistent with its role in regulating central metabolism rather than conferring acid tolerance. Nevertheless, pH-dependent interactions between thermal and essential oil stresses warrant further investigation, particularly in food matrices where pH varies considerably.

## 5 Conclusions and future work

In summary, parallel whole-genome screens of *Salmonella* under combined limonene and heat treatment revealed that central metabolism is the dominant determinant of survival in both planktonic and biofilm states, but that it acts in opposite directions in each: genes whose inactivation aids planktonic survival of heat are essential in biofilms. This lifestyle-dependent inversion means that no single genetic target is likely to be equally effective against both states, and that decontamination strategies may need to be matched to the physiological form in which *Salmonella* is most likely to persist. The convergence of central-metabolism, electron-transport and oxidative-stress genes across conditions further points to oxidative damage as a shared route of killing, offering candidate targets for combinations designed to potentiate essential oils and heat. A principal limitations should be noted: validation of the biofilm determinants was constrained because *crp* and *cyaA* are themselves essential for biofilm formation, preventing straightforward deletion-mutant confirmation in the biofilm state, and the CFU-based validation cannot distinguish killing from entry into the viable but non-culturable (VBNC) state. More broadly, parallel screening across bacterial lifestyles offers a way to anticipate how susceptibility shifts with physiological state, an increasingly important consideration as multi-compound treatments become key to food safety.

## CRediT authorship contribution statement

**Emma R. Holden:** Writing – review & editing, Writing – original draft, Visualization, Validation, Resources, Methodology, Investigation, Formal analysis, Data curation, Conceptualization. **Silvia Guillén:** Writing – review & editing, Validation, Methodology, Investigation, Conceptualization. **Muhammad Yasir:** Writing – review & editing, Methodology, Investigation. **Natalia Perez-Moral:** Writing – review & editing, Methodology, Investigation. **Arjan Narbad:** Writing – review & editing, Resources. **Alfredo Palop:** Writing – review & editing, Supervision, Resources, Methodology, Investigation. **Mark A. Webber:** Writing – review & editing, Supervision, Funding acquisition. **Enriqueta Garcia-Gutierrez:** Writing – review & editing, Writing – original draft, Validation, Supervision, Resources, Project administration, Methodology, Investigation, Funding acquisition, Conceptualization.

## Funding

All sequencing and functional genomics work was supported by the Biotechnology and Biological Sciences Research Council (BBSRC), UK; ERH was funded by the BBSRC Institute Strategic Programme Grant Microbes in the Food Chain BB/R012504/1 and its constituent project BBS/E/F/000PR10349. This research was partly funded with project PID2023-149211OB-C31 by the Spanish Ministry of Science, Innovation and Universities (MCIU) and the Spanish Agency for Research (AEI). EGG is also funded by a Beatriz Galindo scholarship from the Spanish Ministry of Universities (BG22/00060).

## Declaration of competing interest

The authors declare the following financial interests/personal relationships which may be considered as potential competing interests: Enriqueta Garcia-Gutierrez reports financial support was provided by Universidad Politécnica de Cartagena. Emma Holden reports financial support was provided by Quadram Institute. If there are other authors, they declare that they have no known competing financial interests or personal relationships that could have appeared to influence the work reported in this paper.

## Data Availability

Sequence data from TraDIS-*Xpress* experiments in this study has been deposited in ArrayExpress under the accession number E-MTAB-17304. The authors confirm all supporting data, code and protocols have been provided within the article or through supplementary data files. Additional data is available on request.
