# Supplementary material for: Genome-wide screening reveals opposing roles for central metabolism in planktonic and biofilm survival of Salmonella under combined limonene and heat treatment
Source: Biofilm. 2026 Oct 29;12:100406. doi: 10.1016/j.bioflm.2026.100406 (PMC13744872; doi:10.1016/j.bioflm.2026.100406)
Supplement: Supplementary file 1 [file mmc2.docx]

**Supplementary information**

### **Supplementary Figure 1**: Particle size distribution of the prepared nanoemulsion measured using a Universal Liquid Module with water as the carrier fluid. The differential volume (%) is plotted as a function of particle size (µm). The curves represent three independent measurement runs, showing a consistent submicron size distribution with a mean particle size of approximately 0.77 µm.

**Supplementary Figure S2.** Intracellular ROS quantification by DCFH-DA fluorescence in *S.* Typhimurium 14028S. Planktonic cells were exposed to 20 µM DCFH-DA (37 °C, 30 min, dark), washed, and exposed to different treatments: 37 °C (untreated control), 37 °C with 0.2 µL/mL free limonene (Lim), 54 °C, 54 °C with 0.2 µL/mL free limonene, 56 °C, and 56 °C with 0.2 µL/mL free limonene. Hydrogen peroxide (0.1 mM, 37 °C) was included as a positive control to validate probe responsiveness. LB (negative control) confirms the absence of background fluorescence without the probe, and LB + DCFH-DA 20 µM (free probe in medium, no cells and no wash) confirms probe functionality, the effect of the probe and detector sensitivity. Fluorescence was measured at Ex 485 nm / Em 530 nm using a Tecan Infinite 200 PRO microplate reader. Bars represent the mean ± SD of three technical replicates from two biological replicates. Statistical analysis: one-way ANOVA with Tukey HSD post-hoc test; *p < 0.05, ***p < 0.001, ns = not significant.
